# NMR-Based Chemical Profiling, Isolation and Evaluation of the Cytotoxic Potential of the Diterpenoid Siderol from Cultivated *Sideritis euboea* Heldr.

**DOI:** 10.3390/molecules25102382

**Published:** 2020-05-20

**Authors:** Ekaterina-Michaela Tomou, Maria V. Chatziathanasiadou, Paschalina Chatzopoulou, Andreas G. Tzakos, Helen Skaltsa

**Affiliations:** 1 Department of Pharmacognosy & Chemistry of Natural Products, School of Pharmacy, National and Kapodistrian University of Athens, Panepistimiopolis, Zografou, 15771 Athens, Greece; ktomou@pharm.uoa.gr; 2 Department of Chemistry, Section of Organic Chemistry and Biochemistry, University of Ioannina, 45110 Ioannina, Greece; m.chatziathanasiadou@gmail.com; 3 Hellenic Agricultural Organization DEMETER, Institute of Breeding and Plant Genetic Resources, IBPGR, Department of Medicinal and Aromatic Plants, Thermi, 57001 Thessaloniki, Greece; xatzlin@yahoo.gr

**Keywords:** cultivated *Sideritis euboea*, siderol, MTT assay, cancer cell lines, NMR

## Abstract

Diterpenes are characteristic compounds from the genus *Sideritis* L., possessing an array of biological activities. Siderol is the main constituent of the *ent*-kaurene diterpenes in *Sideritis* species. In order to isolate the specific compound and evaluate for the first time its cytotoxic activity, we explored the dichloromethane extract of cultivated *Sideritis euboea* Heldr. To track the specific natural bioactive agent, we applied NMR spectroscopy to the crude plant extract, since NMR can serve as a powerful and rapid tool both to navigate the targeted isolation process of bioactive constituents, and to also reveal the identity of bioactive components. Along these lines, from the rapid 1D ^1^H NMR spectrum of the total crude plant extract, we were able to determine the characteristic proton NMR signals of siderol. Furthermore, with the same NMR spectrum, we were able to categorize several secondary metabolites into chemical groups as a control of the isolation process. Therefore, this non-polar extract was explored, for the first time, revealing eleven compounds—one fatty acid ester; 2-(*p*-hydroxyphenyl)ethylstearate (**1**), three phytosterols; β-sitosterol (**2**), stigmasterol (**3**), and campesterol (**4**); one triterpenoid; ursolic acid (**5**), four diterpenoids; siderol (**6**), eubol (**7**), eubotriol (**8**), 7-epicandicandiol (**9**) and two flavonoids; xanthomicrol (**10**) and penduletin (**11**). The main isolated constituent was siderol. The antiproliferative potential of siderol was evaluated, using the MTT (3-(4,5-Dimethylthiazol-2-yl)-2,5-Diphenyltetrazolium Bromide) assay, on three human cancer cell lines DLD1, HeLa, and A549, where the IC_50_ values were estimated at 26.4 ± 3.7, 44.7 ± 7.2, and 46.0 ± 4.9 μΜ, respectively. The most potent activity was recorded in the human colon cancer cell line DLD1, where siderol exhibited the lowest IC_50_. Our study unveiled the beneficial potential of siderol as a remarkable cytotoxic agent and the significant contribution of NMR spectroscopy towards the isolation and identification of this potent anticancer agent.

## 1. Introduction

Over the last decades, natural products have been at the forefront as major drug sources in cancer and the explosion of knowledge in this area has brought forward new drug discoveries and therapeutic developments. Diterpenes represent about 20% of the terpenes and exert a broad range of significant biological activities, including antimicrobial, antioxidant, anti-inflammatory, anti-HIV, anticholinesterase, and antiproliferative activity [1,2,3,4,5,6].

In recent years, the genus *Sideritis* is one of the most economically explored in the Lamiaceae family, as its commercial need is continuously on the rise in the global market. Therefore, in the last few years, there is an effort to cultivate these important medicinal species in order to meet the commercial need and provide high-quality products. Furthermore, *Sideritis* species are considered as remarkable medicinal plants with great pharmacological activities, attributed to their rich phytochemical profile [4,7]. The European Medicine Agency (EMA) monograph mentions the infusion of *Sideritis* species (*S. scardica* Griseb.; *S. clandestina* (Bory & Chaub.) Hayek; *S. raeseri* Boiss. & Heldr.; and *S. syriaca* L.), known as Greek mountain tea, as traditional medicine for the relief of mild gastrointestinal discomfort and against the common cold [7]. Currently, a clinical study also revealed that *S. scardica* (Greek mountain tea) enhanced the memory capacity and the mood in healthy older adults [8].

In the context of chemotaxonomy, the genus *Sideritis* L. (Lamiaceae) comprises more than 150 species, which are mainly distributed in the Mediterranean area and are classified into two subgenera—*Sideritis* and *Leucophae*. The subgenus *Sideritis* is divided into four sections, *Hesiodia* (Moench) Bentham, *Empedoclia* (Rafin) Bentham, *Burgsdorfia* (Moench) Briquet, and *Sideritis* L. [1,9,10]. Diterpenes are characteristic constituents of genus *Sideritis*, which are classified according to their structure to different types (*ent*-kaurene, labdane, atisene, pimarane, beyerane, trachilobane, and rosane) [9]. Previous chemotaxonomic studies mentioned that the *ent*-kaurene type derivatives are exclusively biosynthesized in species of the Eastern and Central Mediterranean area (Turkey, Greece, and Italy), while species growing in the Western Mediterranean area and in Macaronesia present various types [9,10]. Specifically, the Mediterranean species are characterized by tetracyclic diterpenes with *ent*-kaurene skeleton derivatives as major compounds. Siderol is a well-known *ent*-kaurene diterpenoid of the *Sideritis* spp [9]. Even though siderol is considered as a common major constituent of the genus *Sideritis*, few biological studies have been conducted, revealing only its antimicrobial, antiviral, antioxidant, and anticholinesterase activity [11,12,13,14,15]. Up-to-now, its potential to serve as an anticancer agent has not been explored or exploited. 

Over the last years, NMR spectroscopic applications have contributed to the design of strategies and the discovery of new bioactive compounds from complex matrices like plants, permitting the direct analysis of complex mixtures and crude extracts. In a previous study [16], we reported the bioactive secondary metabolites from the methanol extract of aerial parts of cultivated *S. euboea* Heldr., using NMR analysis. 

In continuation of our phytochemical investigations into cultivated *Sideritis* species, herein, we report on the isolation and identification of phytochemicals from the non-polar extract from the aerial parts of the specific plant. Precisely, the current study was designed to guide the isolation of siderol and evaluate its cytotoxic activity since this remained elusive. In an effort to identify this bioactive secondary compound from the crude extract, we applied NMR spectroscopy in the early stage of the experimental process. Afterward, we examined the effect of isolated siderol against three human cancer cell lines—DLD1, HeLa, and A549. As far as we know, this is the first study to report on the anticancer potential of siderol.

## 2. Results and Discussion

**Figure 1:**
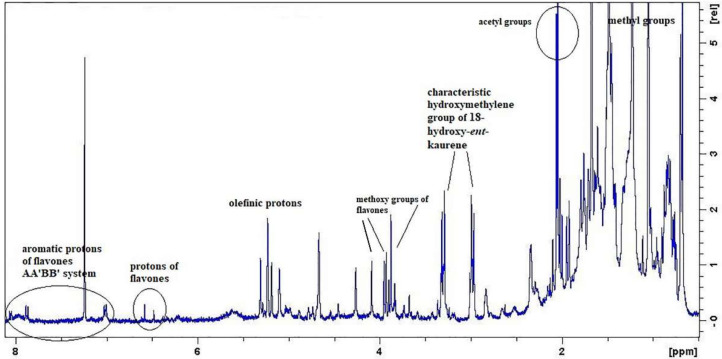
^1^H-NMR spectrum of the dichloromethane extract from cultivated *S. euboea*.

The present study was focused on the isolation and identification of the bioactive compound, siderol from the non-polar extract of the cultivated *S. euboea*. To avoid time-consuming and cost-ineffective steps in the isolation process, we utilized simple and rapid 1D ^1^H-NMR spectroscopy as a powerful tool to rapidly guide the isolation process and screening of the chemical components present in the complex crude plant extract. Considering the fact that *ent*-kaurene derivatives are mainly non-polar compounds, we decided to investigate the dichloromethane extract of the plant. At the onset of the experimental process, we applied 1D ^1^H-NMR spectroscopy in the crude extract so as to monitor the presence of siderol and then, to identify the presence of other secondary metabolites. The ^1^H-NMR spectrum of the dichloromethane extract unveiled the characteristic proton NMR pattern derived from siderol. Furthermore, the evaluation of the NMR spectrum of the crude extract revealed signals arising from phytosterols/triterpenoids, 18-hydroxy-*ent*-kaurene derivatives, and methoxylated flavones (Figure 1). In this initial stage of the isolation process, the application of NMR spectroscopy was considered as a powerful tool to aid in the a) tracking of the targeted bioactive compound, b) in understanding the chemical categories of the compounds present in the crude extract better, and c) in guiding the isolation process. Then, the crude extract was fractionated by using Column Chromatography (CC) techniques. During the isolation process, the obtained fractions from each CC were monitored by Thin Layer Chromatography (TLC) and were properly grouped. Then, the ^1^H-NMR spectra of the interesting groups were recorded, and based on these spectra, the further isolation steps were guided. This strategy was followed for the isolation and purification of the desired bioactive compounds. This isolation process is time- and cost-effective, as failed isolation steps are disregarded in the overall isolation pipeline. The structure elucidation of all the isolated compounds was undertaken by NMR spectroscopy, and their spectroscopic data were compared to those formerly published.

**Figure 2:**
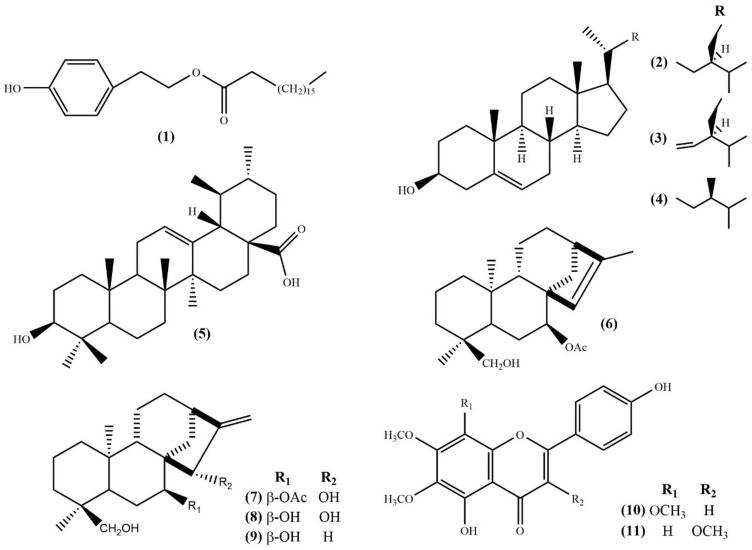
Structures of isolated compounds **1**–**11**.

As a result, the chemical investigation of the dichloromethane extract of cultivated *S. euboea* aerial parts revealed in total **11** compounds, including one fatty acid ester, 2-(*p*-hydroxyphenyl)ethylstearate (**1**) [17]; three phytosterols, namely *β*-sitosterol (**2**) [18], stigmasterol (**3**) [18], and campesterol (**4**) [19]; one triterpenoid, ursolic acid (**5**) [20]; four diterpenoids, i.e. siderol (**6**) [21], eubol (**7**) [22], eubotriol (**8**) [22], 7-epicandicandiol (**9**) [23] and two flavonoids, xanthomicrol (**10**) [24] and penduletin (**11**) [25] (Figure 2). 

Based on the literature data, compound **1** had not been previously isolated in the genus *Sideritis*. The phytosterols and the triterpenoid (compounds **2**–**5**) have been previously reported in this genus and in Lamiaceae family; however, they are reported here, as components of *S. euboea* for the first time. At this point, we should notice that the olefinic protons of the specific compounds, as well as their methyl groups, were depicted in the ^1^H-NMR spectrum of the crude plant extract (Figure 1). 

**Figure 3:**
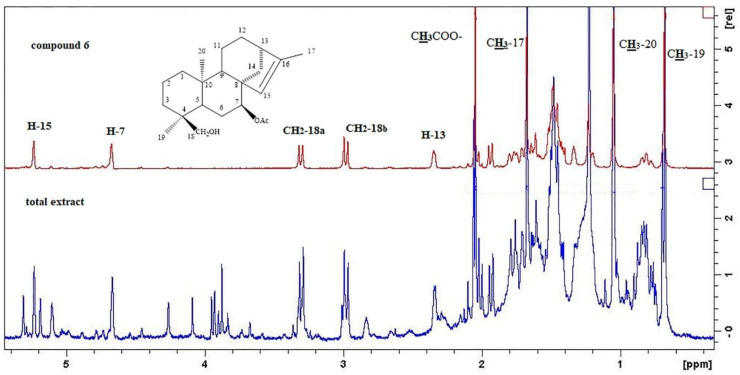
^1^H-NMR spectrum of the crude dichloromethane extract of cultivated *Sideritis euboea* and of the isolated compound **6** from the same extract.

Previous phytochemical studies reported few diterpenoids from the wild populations of *S. euboea*, including isofoliol, siderol, sideridiol, sideroxol, epoxysiderol, eubol, and eubotriol [10,11,26]. It is remarkable to point out that siderol was isolated from two main fractions in a large amount, so we could consider that it serves as a major component of the dichloromethane extract. In order to show the identification of siderol from the crude extract, we compared, herein, both the ^1^H-NMR spectra of the total extract and the isolated compound **6** (Figure 3). Indeed, in the ^1^H-NMR spectrum of the crude extract, we were able to locate the characteristic proton signals of siderol at δ 5.23 (1H, s, H-15), 4.66 (1H, t= 2.0 J, H-7), 3.30 (1H, d=10.2 J, CH_2_-18a), 2.98 (1H, d=10.2 J, CH_2_-18b), 2.34 (1H, m, H-13), 2.08 (3H, s, CH_3_COO), 1.67 (3H, s, CH_3_-17), 1.04 (3H, s, CH_3_-20), and 0.68 (3H, s, CH_3_-19) (Figure 3, Figures S1–S5). In addition, we were able to confirm the presence of eubol and eubotriol in the extract, which were firstly discovered in 1977 in the wild species [22]. Furthermore, compound **9** was also identified in the specific genus, but not in the species *S. euboea*. 

Fraga (2012) divided the *Sideritis* species of Europe into four groups, based on the presence of diterpenes and triterpenes. In particular, the first group includes the *Sideritis* spp. that contain triterpenes or sterols, but not diterpenes; the second group consists of species that have major labdane type diterpenes; the third group is characterized by tetracyclic *ent*-kaurene type diterpenes, including the Mediterranean species, mainly from *Sideritis* and *Empedoclia* sections; the last group is formed of different types of diterpenes. Based on the presence of four *ent*-kaurene skeleton diterpenes in our results, we could classify the cultivated *S. euboea* to the third group. Our data also revealed the existence of one triterpene and three phytosterols, as a corollary of this, we could surmise that the cultivation method could influence the phytochemical profile of the plant.

Considering the methoxylated flavones, compounds **10** and **11** were previously mentioned in the genus *Sideritis* L. several times; however, they were isolated as components of *S. euboea* for the first time [9,10,27]. In the ^1^H-NMR spectrum of the crude extract, we were also able to identify characteristic proton signals of the two methoxylated flavones (Figure 1). In particular, in the aromatic region of the spectrum, we could observe the two AA′ΒΒ′ systems of the two rings B of each flavone and the aromatic protons of the ring C or A of each flavone, respectively. In the middle region of the spectrum, we could also recognize their methoxy groups in the range δ 4.09–3.87. 

**Figure 4:**
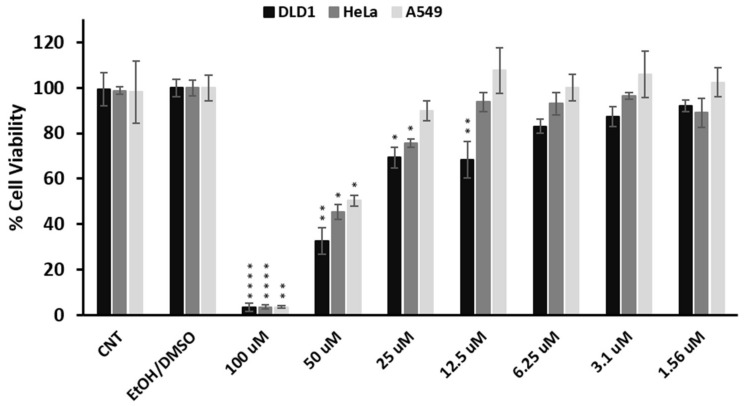
Cell viability of DLD1, HeLa, and A549 cells line under treatment with siderol. Cells were treated with the indicated concentrations of siderol for 72 h, and the viability was estimated by the MTT assay. CNT (control) corresponds to the untreated cells and EtOH/DMSO to the highest volume (0.1% *v/v*) of the ethanol: dimethyl sulfoxide (50:50 *v/v*) solution, which was added to the cells. Statistical analysis was conducted with the Dunnett’s test. **** *p* < 0.0001, ** *p* < 0.01, * *p* < 0.1.

Accumulating studies have demonstrated the antiproliferative activity of *ent*-kaurene diterpenes on various human cancer cell lines [14,28,29,30]. To the best of our knowledge, the potential anticancer activity of siderol remained elusive. Thus, in order to explore its potential cytotoxic effect in vitro, the viability of three cancer cell lines (DLD1, HeLa, A549) in the presence of various concentrations (0–100 μΜ) of siderol for 72 h was assessed through the MTT assay. Interestingly, concentrations at 100 and 50 μΜ inhibited the growth in all three cell lines significantly (Figure 4). The IC_50_ values of siderol in DLD1, HeLa, and A549 cells were 26.4 ± 3.7 μΜ, 44.7 ± 7.2, and 46.0 ± 4.9 μΜ, respectively. The diterpenoid showed an enhanced cytotoxic effect on the DLD1 cell line compared to HeLa and A549 cells, where siderol’s IC_50_ values were almost two-fold the IC_50_ value in the DLD1. The magnitude of the IC_50_ values, and especially the one exhibited by siderol in the DLD1 cell lines is quite low amongst the values that are usually demonstrated by phytochemical compounds. For example, these IC_50_ values are lower compared to those presented by the widely-studied terpene α-pinene, which shows a lower inhibitory effect in HepG2, A549, PC-12, and MCF-7 cell lines [31], or they are in the same range with the IC_50_ values displayed by the prominent flavonoid quercetin [32]. On the other hand, the IC_50_ values presented by siderol are higher compared to those of established chemotherapeutic drugs such as fluorouracil (2.95, 17.21, and 8.13 μM in DLD1 [33], HeLa [34], and A549 [34] cells, respectively) and the plant-derived paclitaxel (133, 2.6, and 4.1 nM in DLD1 [33], HeLa [35], and A549 [35] cells, respectively). Thus, siderol has great potential to be exploited as a potent anticancer agent, and its derivatization or formulation should be considered in order to enhance even more its cytotoxic efficacy.

**Figure 5:**
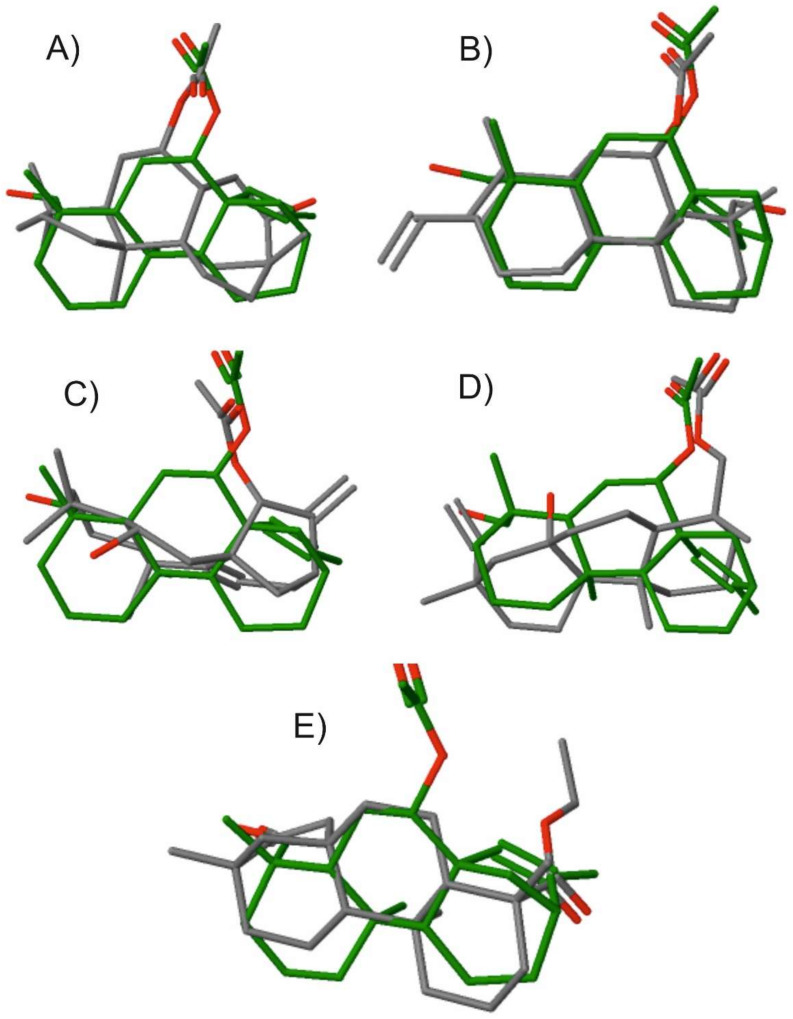
Superposition of siderol (colored in green) with different identified bioactive compound hits (colored in grey). (**A**) (4a*S*,6*S*,6a*S*,8*S*,9*R*,11a*S*,11b*S*)-8-hydroxy-4,4,9,11b-tetramethyltetradecahydro-9,11a-methanocyclohepta[a]naphthalen-6-yl acetate (CHEMBL494391), (**B**) (4a*S*,4b*R*,8*R*,8a*R*,9*R*,10a*R*)-8-(hydroxymethyl)-1,4b,8-trimethyl-2-vinyl-3,4,4a,4b,5,6,7,8,8a,9,10,10a-dodecahydrophenanthren-9-yl acetate (CHEMBL482794), (**C**) (4a*R*,5*S*,6a*S*,7*R*,9*R*,11b*R*)-5-hydroxy-4,4,11b-trimethyl-8-methylene-1,2,3,4,4a,5,6,7,8,9,10,11b-dodecahydro-6a,9-methanocyclohepta[a]naphthalen-7-yl acetate (CHEMBL448113) and (**D**) ((1*R*,4a*S*,4b*R*,7*S*,8a*S*,10a*R*)-8a-hydroxy-1,4a,7-trimethyl-7-vinyltetradecahydrophenanthren-1-yl)methyl acetate (CHEMBL509521), and (**E**) ethyl (4*R*,4a*S*,6a*R*,9*S*,11a*R*,11b*S*)-4,9,11b-trimethyl-8-oxotetradecahydro-6a,9-methanocyclohepta[a]naphthalene-4-carboxylate (CHEMBL455441).

In order to evaluate the potential similarity of the isolated chemotype of siderol with other compounds with known bioactivity in the CHEMBL database, we conducted a 3D-based virtual screening [36]. The highest hits, going from the highest-ranking to the lowest, were compounds (4a*S*,6*S*,6a*S*,8*S*,9*R*,11a*S*,11b*S*)-8-hydroxy-4,4,9,11b-tetramethyltetradecahydro-9,11a-methanocyclohepta[a]naphthalen-6-yl acetate (CHEMBL494391), (4a*S*,4b*R*,8*R*,8a*R*,9*R*,10a*R*)-8-(hydroxymethyl)-1,4b,8-trimethyl-2-vinyl-3,4,4a,4b,5,6,7,8,8a,9,10,10a-dodecahydrophenanthren-9-yl acetate (CHEMBL482794), (4a*R*,5*S*,6a*S*,7*R*,9*R*,11b*R*)-5-hydroxy-4,4,11b-trimethyl-8-methylene-1,2,3,4,4a,5,6,7,8,9,10,11b-dodecahydro-6a,9-methanocyclohepta[a]naphthalen-7-yl acetate (CHEMBL448113), ((1*R*,4a*S*,4b*R*,7*S*,8a*S*,10a*R*)-8a-hydroxy-1,4a,7-trimethyl-7-vinyltetradecahydrophenanthren-1-yl)methyl acetate (CHEMBL509521), and ethyl (4*R*,4a*S*,6a*R*,9*S*,11a*R*,11b*S*)-4,9,11b-trimethyl-8-oxotetradecahydro-6a,9-methanocyclohepta[a]naphthalene-4-carboxylate (CHEMBL455441). The relevant 2D structures and nomenclature of these compounds are illustrated in the Supplementary Information (Figure S6). The structure superposition of siderol with these five hits is illustrated in Figure 5 on the frame of which a rather good 3D alignment was recorded. Providing that similar chemotypes could instruct and code for similar bioactivity, we then explored the bioactivity that has been recorded for the identified hits. The first four compounds have been evaluated and identified to possess antiviral, antifungal, antitrypanosomal, and antifungal activity, respectively. Interestingly, the last compound ethyl (4*R*,4a*S*,6a*R*,9*S*,11a*R*,11b*S*)-4,9,11b-trimethyl-8-oxotetradecahydro-6a,9-methanocyclohepta[a]naphthalene-4-carboxylate (CHEMBL455441) has illustrated antiproliferative activity against the human colon cancer cell line HCT116. The tracked chemical similarity of siderol with other chemotypes bearing a diverse array of bioactivities highlights the potential of this compound to illustrate diverse bioactivity that has to be exploited. Of interest is that overlapping anticancer bioactivity and specifically in colon cancer was also reported for another compound bearing high structural similarity with siderol. These results capitalize on the bioactive potential that siderol bears that should be further exploited in the future, especially in colon cancer. Such studies will benefit from the methodology we report herein on the large yield isolation of siderol from cultivated *S. euboea*. These studies are in progress in our labs. 

## 3. Materials and Methods 

### 3.1. General Procedures

1D and 2D NMR spectra were recorded in CDCl_3_ on Bruker DRX 400 instrument (Bruker Biospin Gmbh, Rheinstetten, Germany) at 295 K. Chemical shifts are given in ppm (δ) and were referenced to the solvent signals at 7.24 and 77.0 ppm. COSY (COrrelation SpectroscopΥ), HSQC (Heteronuclear Single Quantum Correlation), HMBC (Heteronuclear Multiple Bond Correlation), and NOESY (Nuclear Overhauser Effect SpectroscopY) (mixing time 950 ms) experiments were performed using standard Bruker microprograms. Vacuum liquid chromatography (VLC): silica gel 60H (Merck, Art. 7736). Column chromatography (CC): silica gel (Merck, Art. 9385), gradient elution with the solvent mixtures indicated in each case. Fractionation was always monitored by TLC silica gel 60 F- 254 (Merck, Art. 5554) with visualization under UV (254 and 365 nm) and spraying with vanillin-sulfuric acid reagent (vanillin Merck, Art. No. S26047 841). All obtained extracts, fractions, and isolated compounds were evaporated to dryness in a vacuum under low temperature and then were put in activated desiccators with P_2_O_5_ until their weights had stabilized. This procedure allows the elimination of moisture from the samples that might influence pre-saturation performance and then lead to an intense water signal in the ^1^H-NMR spectra, making it difficult to observe near signals.

### 3.2. Plant Material

Aerial parts of *S. euboea* Heldr. were collected from a cultivated population (under organic farming conditions) in HAO DEMETER (Institute of Breeding and Plant Genetic Resources) in July 2017. The sample was authenticated by Dr P. Chatzopoulou; a voucher specimen was deposited in the herbarium of Aromatic and Medicinal Plant Department (code 19-17). The plant material was dried for 10 days at room temperature, and then powdered in a specific pulverization machine without freezing.

### 3.3. Extraction and Isolation

The air-dried powdered plant material (0.30 kg) was extracted at room temperature with cyclohexane, dichloromethane and methanol, successively and concentrated to dryness to yield residues of 9.2 g, 4.6 g and 25.3 g, respectively. Afterward, a part of the dichloromethane extract (1.3 g) was pre-fractionated by VLC over silica gel (10.0 cm × 6.0 cm), using as eluent mixtures of increasing polarity (cyclohexane:dichloromethane:ethyl acetate:methanol) to yield finally 12 fractions of 500 mL (A–M). Fractions B (80 mg; eluted with CH_2_Cl_2_:EtOAc 95:5), C (166.2 mg; eluted with CH_2_Cl_2_:EtOAc 90:10), D (100 mg; eluted with CH_2_Cl_2_:EtOAc 80:20), E (145.1 mg; eluted with CH_2_Cl_2_:EtOAc 70:30) were separately submitted to CC over silica gel (CyHex:CH_2_Cl_2_:EtOAc 100:0:0 to 0:0:100) and afforded nine compounds, as follows: Fr. B: **1** (6.2 mg), **2** (1.1 mg) and **3** (1.2 mg); Fr. C: **4** (5.5 mg) and **5** (2.1 mg); Fr. D: **6** (15.0 mg); Fr. E: **6** (6.8 mg), a mixture of compounds **6** and **7** (9.3 mg), **8** (1.8 mg) and **9** (1.0 mg). In addition, fractions H (60.4 mg; eluted with CH_2_Cl_2_:EtOAc 40:60) and I (20.8 mg; eluted with CH_2_Cl_2_:EtOAc 30:70) were separately submitted to CC over silica gel (CyHex:CH_2_Cl_2_:(CH_3_)_2_CO 100:0:0 to 0:0:100) and gave compounds **10** (1.7 mg) and **11** (1.2 mg), respectively.

### 3.4. Cell Cultures

DLD1 colon cancer cells were cultured in McCoy’s 5A medium (Hyclone, Logan, UT, USA). A549 lung cancer and HeLa cervical cancer cells were cultured in low glucose DMEM (Dulbecco’s Modified Eagle’s medium) (Sigma, St. Louis, MO, USA). All media were supplemented with 10% FBS (Gibco, Carlsbad, CA, USA), 100 U/mL Penicillin, and 100 μg/mL Streptomycin (Gibco). The cell cultures were maintained at 37 °C in a humidified atmosphere of 5 % CO_2_.

### 3.5. Viability Assay

DLD1, HeLa, and A549 cell lines were seeded into 96-well plates at densities of 3000, 4000, and 1500 cells/well, respectively. The next day the cells were treated with various concentrations of siderol in triplicates (0–100 μΜ) for 72 h. Siderol was dissolved in EtOH:DMSO (50:50 *v/v*) due to its low aqueous solubility. However, the final volume of DMSO did not exceed 0.5% (*v/v*) each time and it was well-tolerated in all three cell lines. After the incubation period, 10 μL of MTT solution (5 mg/mL in PBS) was added to each well and left to 37 °C for another 4 h. Finally, the supernatant of each well was removed, and 100 μL of DMSO was added to dissolve the formazan crystals. The absorbance was measured at 540 nm via an ELISA microplate reader (Awareness Technology Inc, Westport, CT, USA). The IC_50_ value of siderol for each cell line was estimated from three independent experiments using GraphPad Prism 8 (non-linear fitting). Statistical analysis was also carried out using GraphPad Prism 8. The treatment effects were compared to the control using the Dunnett’s test. The *p*-value was considered at 5% level of significance.

## 4. Conclusions

The species *S. euboea* is a narrow endemic and threatened perennial herb with a small distribution area that should not be collected from the field. Therefore, the present study deals with the first chemical investigation of the dichloromethane extract of cultivated *S. euboea*. Plants sold in local markets should come from standard cultivations. Thus, the identification of their phytochemical profiles should be thoroughly studied. At the first step, our results showed that the cultivation of *S.euboea* was feasible without affecting its chemical composition, compared to the wild species.

Furthermore, considering that *ent*-kaurene diterpenes have shown great pharmacological activities, we evaluated the natural bioactive compound siderol for its cytotoxic activity. We applied NMR analysis in the crude extract to identify this concrete secondary metabolite, and afterward, we isolated it. The specific strategy, using the 1D NMR application in the total extract enhanced us to determine and isolate the compound from the early stage of the process, to isolate secondary metabolites for the first time in the specific species and then to minimize the time and the costs of the whole analytical procedure with great results. 

This study not only shed light on the non-polar secondary metabolites of cultivated *S. euboea* Heldr., but it also revealed the beneficial anticancer potential of siderol, its main constituent. Due to its remarkable cytotoxic effect, it could serve as a natural product scaffold-core structure for the development of more active derivatives. Further investigation of the structure-activity relationship of these derivatives, as well as the interaction with their molecular targets, need to be undertaken for further research as anticancer agents. 

## Supplementary Materials

The following are available online, Figure S1: ^1^H-NMR spectrum of compound **6** (CDCl_3_), Figure S2: ^1^H–^1^H COSY 2D spectrum of compound **6** (CDCl_3_), Figure S3: ^1^H–^13^C HSQC 2D spectrum of compound **6** (CDCl_3_), Figure S4: ^1^H–^13^C HMBC 2D spectrum of compound **6** (CDCl_3_), Figure S5: ^13^C-NMR spectrum of compound **6** (CDCl_3_), Figure S6: 2D Structures of (1) (4a*S*,6*S*,6a*S*,8*S*,9*R*,11a*S*,11b*S*)-8-hydroxy-4,4,9,11b-tetramethyltetradecahydro-9,11a-methanocyclohepta[a]naphthalen-6-yl acetate [CHEMBL494391], (2) (4a*S*,4b*R*,8*R*,8a*R*,9*R*,10a*R*)-8-(hydroxymethyl)-1,4b,8-trimethyl-2-vinyl-3,4,4a,4b,5,6,7,8,8a,9,10,10a-dodecahydrophenanthren-9-yl acetate [CHEMBL482794], (3) (4a*R*,5*S*,6a*S*,7*R*,9*R*,11b*R*)-5-hydroxy-4,4,11b-trimethyl-8-methylene-1,2,3,4,4a,5,6,7,8,9,10,11b-dodecahydro-6a,9-methanocyclohepta[a]naphthalen-7-yl acetate [CHEMBL448113], (4) ((1*R*,4a*S*,4b*R*,7*S*,8a*S*,10a*R*)-8a-hydroxy-1,4a,7-trimethyl-7-vinyltetradecahydrophenanthren-1-yl)methyl acetate [CHEMBL509521] and (5) ethyl (4*R*,4a*S*,6a*R*,9*S*,11a*R*,11b*S*)-4,9,11b-trimethyl-8-oxotetradecahydro-6a,9-methanocyclohepta[a]naphthalene-4-carboxylate [CHEMBL455441].
