# Supplementary material for: NMR-Based Chemical Profiling, Isolation and Evaluation of the Cytotoxic Potential of the Diterpenoid Siderol from Cultivated Sideritis euboea Heldr
Source: Molecules. 2020 May 20;25(10):2382. doi: 10.3390/molecules25102382 (PMC7287962; doi:10.3390/molecules25102382)
Supplement: Supplementary file 1 [file molecules-25-02382-s001.pdf]

NMR-Based Chemical Profiling, Isolation and Evaluation of the Cytotoxic Potential of the Diterpenoid Siderol from Cultivated *Sideritis euboea* Heldr.

Ekaterina-Michaela Tomou[1], Maria V. Chatziathanasiadou[2], Paschalina Chatzopoulou[3], Andreas G. Tzakos[2,*] and Helen Skaltsa[1,*]

[1]Department of Pharmacognosy & Chemistry of Natural Products, School of Pharmacy, National and Kapodistrian University of Athens, Panepistimiopolis, Zografou, 15771, Athens, Greece; ktomou@pharm.uoa.gr; skaltsa@pharm.uoa.gr

[2]Department of Chemistry, Section of Organic Chemistry and Biochemistry, University of Ioannina, 45110, Ioannina, Greece; m.chatziathanasiadou@gmail.com; atzakos@uoi.gr

[3]Hellenic Agricultural Organization DEMETER, Institute of Breeding and Plant Genetic Resources, IBPGR, Department of Medicinal and Aromatic Plants, Thermi, 57001 Thessaloniki, Greece; xatzlin@yahoo.gr

Correspondence: atzakos@uoi.gr (A.G.T.); skaltsa@pharm.uoa.gr (H.S.); Tel./Fax.: +30 2107274593(H.S.)

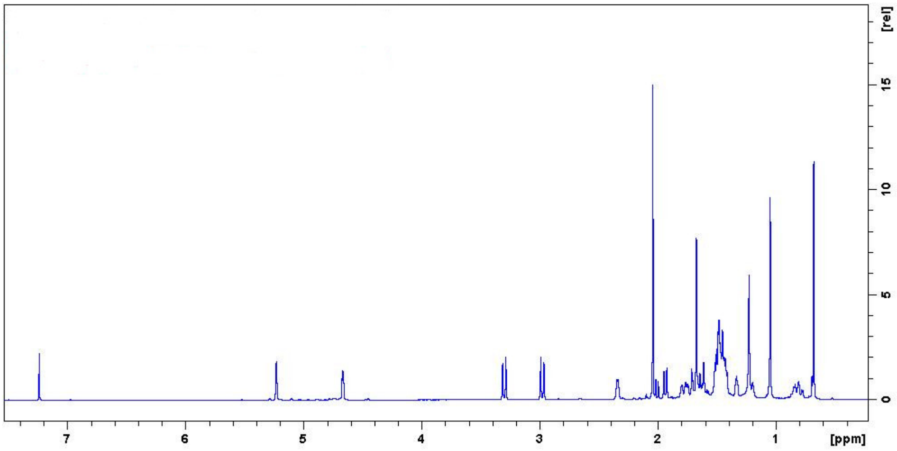

Figure S1. ^{1}H-NMR spectrum of compound **6** ($CDCl_3$, 400 MHz) (T=295 K; number of scans, 16).

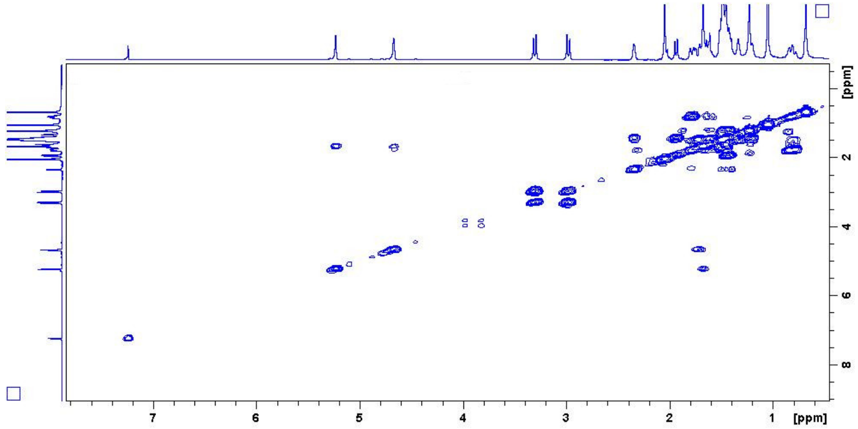

Figure S2. ^{1}H–^{1}H COSY 2D spectrum of compound **6** ($CDCl_3$, 400 MHz) (T=295 K, number of scans, 4).

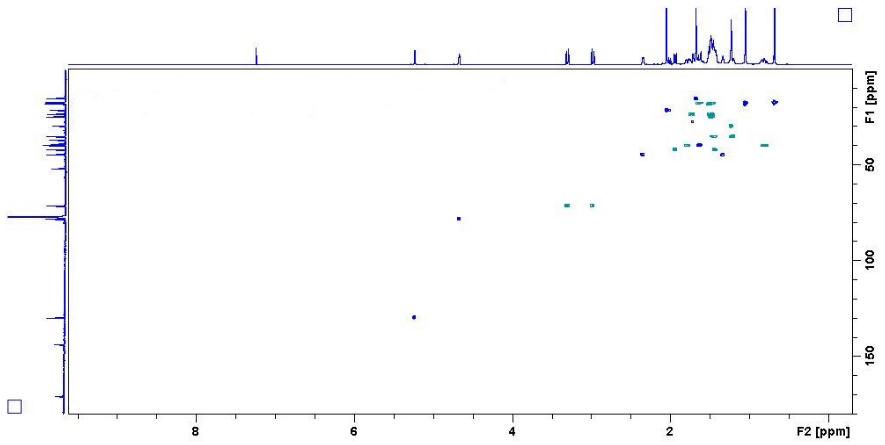

Figure S3. ^{1}H-^{13}C HSQC 2D spectrum of compound **6** ($CDCl_3$, 400 MHz) (T=295 K, number of scans, 20).

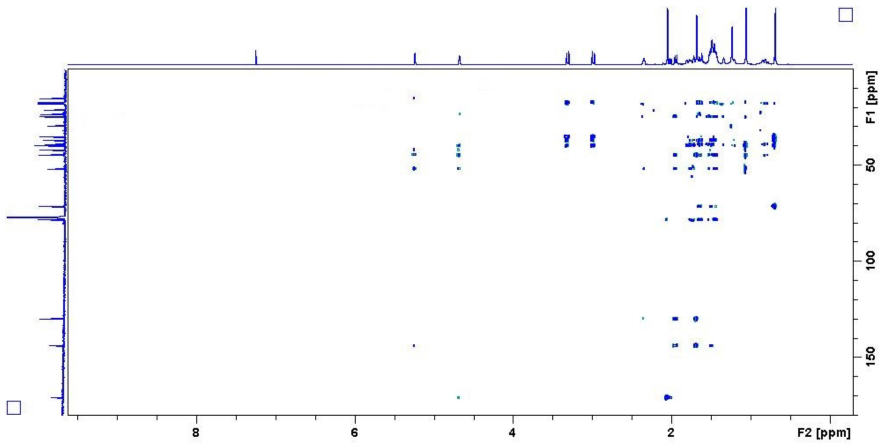

Figure S4. ^{1}H-^{13}C HMBC 2D spectrum of compound **6** ($CDCl_3$, 400 MHz) (T=295 K; number of scans, 48).

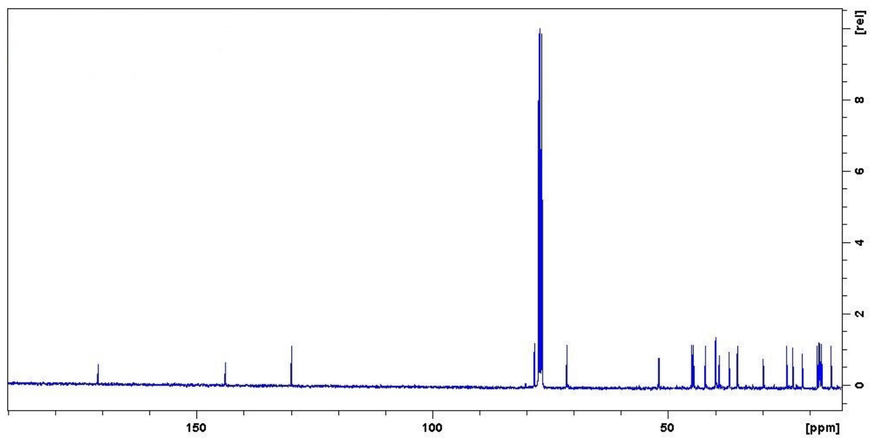

Figure S5. ^{13}C-NMR spectrum of compound **6** ($CDCl_3$, 100.3 MHz) (T=295 K; number of scans, 9024).

(1)

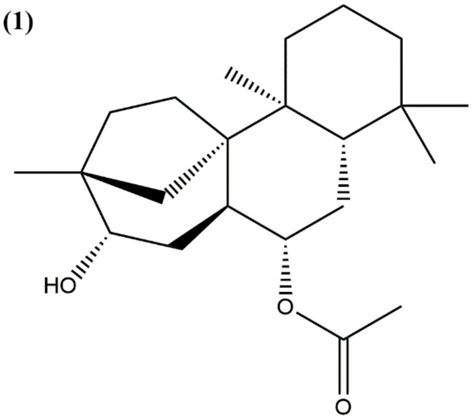

(2)

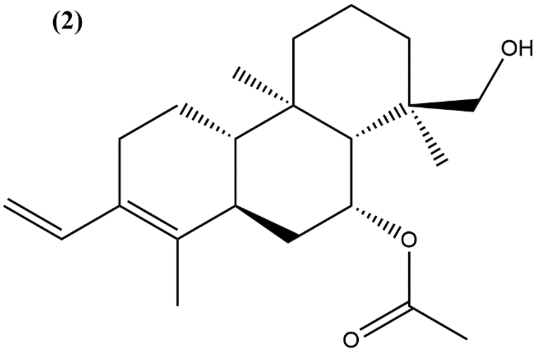

(3)

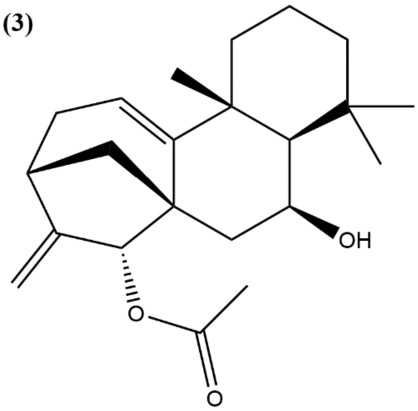

(4)

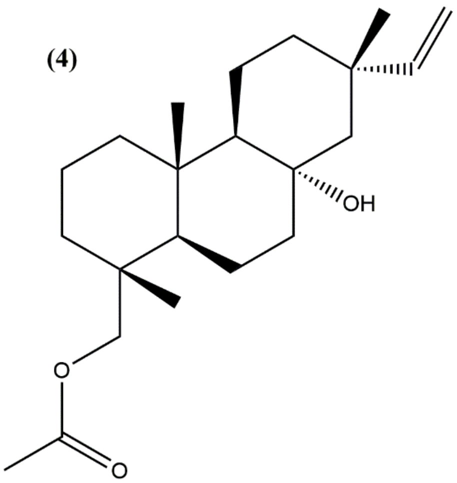

(5)

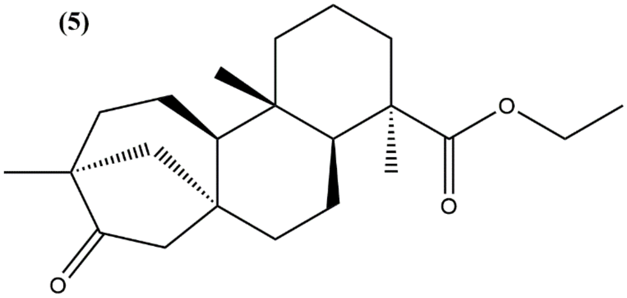

Figure S6. 2D Structures of **(1)** (4a*S*,6*S*,6a*S*,8*S*,9*R*,11a*S*,11b*S*)-8-hydroxy-4,4,9,11b-tetramethyltetradecahydro-9,11a-methanocyclohepta[a]naphthalen-6-yl acetate [CHEMBL494391], **(2)** (4a*S*,4b*R*,8*R*,8a*R*,9*R*,10a*R*)-8-(hydroxymethyl)-1,4b,8-trimethyl-2-vinyl-3,4,4a,4b,5,6,7,8,8a,9,10,10a-dodecahydrophenanthren-9-yl acetate [CHEMBL482794], **(3)** (4a*R*,5*S*,6a*S*,7*R*,9*R*,11b*R*)-5-hydroxy-4,4,11b-trimethyl-8-methylene-1,2,3,4,4a,5,6,7,8,9,10,11b-dodecahydro-6a,9-methanocyclohepta[a]naphthalen-7-yl acetate [CHEMBL448113], **(4)** ((1*R*,4a*S*,4b*R*,7*S*,8a*S*,10a*R*)-8a-hydroxy-1,4a,7-trimethyl-7-vinyltetradecahydrophenanthren-1-yl)methyl acetate [CHEMBL509521] and **(5)** ethyl (4*R*,4a*S*,6a*R*,9*S*,11a*R*,11b*S*)-4,9,11b-trimethyl-8-oxotetradecahydro-6a,9-methanocyclohepta[a]naphthalene-4-carboxylate [CHEMBL455441]
